# Supplementary material for: Deep-learning-derived glaucoma-related endophenotypes enable novel genome-wide genetic and functional discovery
Source: medRxiv. 2025 Nov 6:2025.11.04.25339517. Preprint. [Version 1] doi: 10.1101/2025.11.04.25339517 (PMC12637757; doi:10.1101/2025.11.04.25339517)
Supplement: 1 — Fig. S1. Summary of colocalizing genes (e/sGenes) from the European meta-analysis results across GTEx tissues and retina. Fig. S2. Cross-ancestry GWAS enrichment of specific cell types in ONH and anterior segment. Fig. S3. EUR GWAS enrichment of specific cell types in ONH and anterior segment. Fig. S4. Single-cell expression of genes in various cell types in anterior segment and optic nerve. [file NIHPP2025.11.04.25339517V1-supplement-1.pdf]

medRxiv preprint doi: https://doi.org/10.1101/2025.11.04.25339517; this version posted November 6, 2025. The copyright holder for this preprint (which was not certified by peer review) is the author/funder, who has granted medRxiv a license to display the preprint in perpetuity.
It is made available under a CC-BY 4.0 International license .

Supplementary Figures

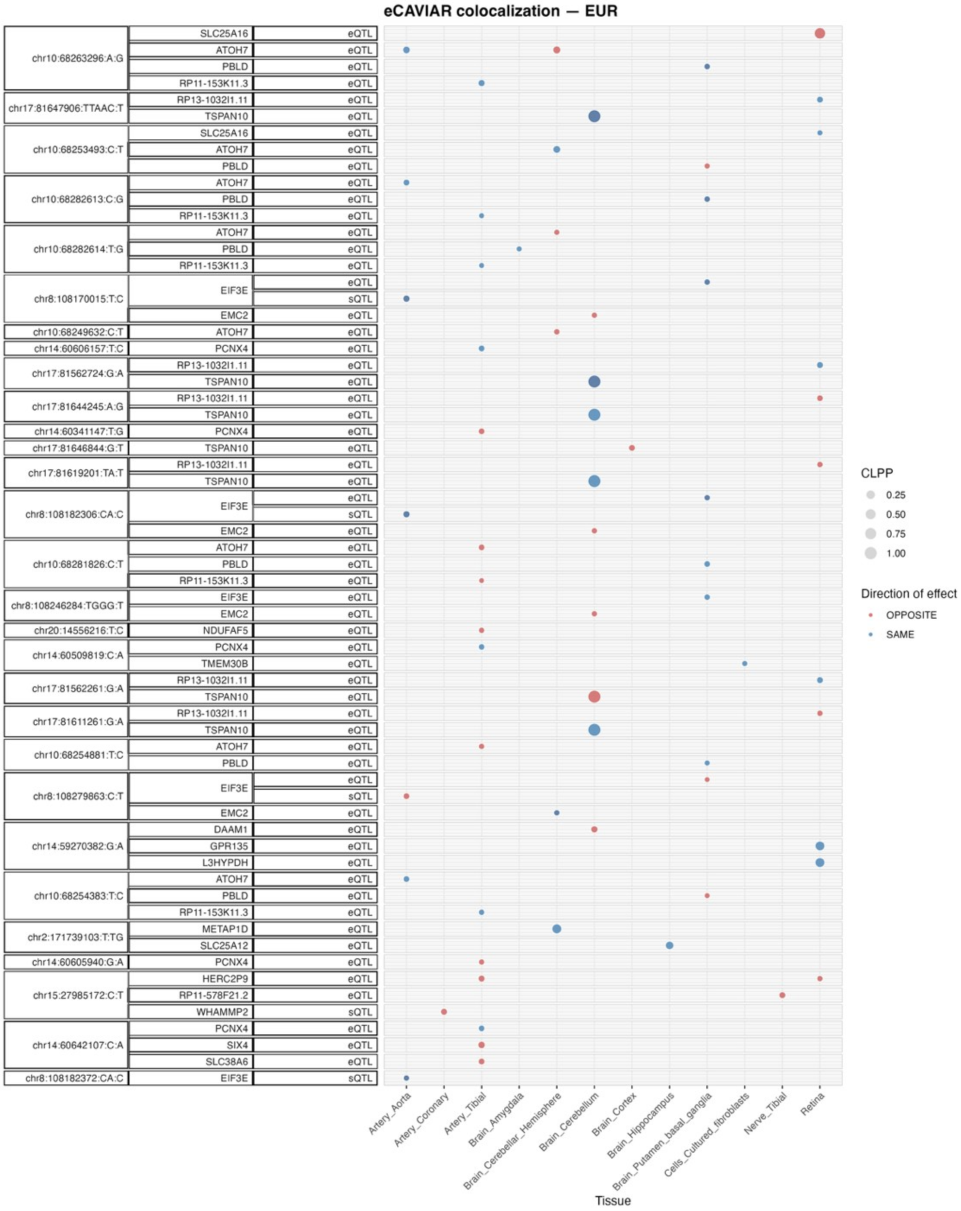

Fig. S1. Colocalizing eQTLs and sQTLs in GTEx tissues and retina with genome-wide significant loci in EUR ML endophenotype meta-analyses. Genes with at least one significant colocalization result (CLPP>0.01) are shown for e/sQTLs tested across selected GTEx tissues and peripheral retina for the genome-wide significant loci from the EUR and the cross-ancestry endophenotype meta-analyses. Bubble size is proportional to the maximum colocalization posterior probability (CLPP) of all e/sVariants tested for the given gene, QTL type and tissue combination. Points are color-coded by the relative direction of effect between the QTL and the GWAS meta-analysis.

medRxiv preprint doi: https://doi.org/10.1101/2025.11.04.25339517; this version posted November 6, 2025. The copyright holder for this preprint (which was not certified by peer review) is the author/funder, who has granted medRxiv a license to display the preprint in perpetuity.
It is made available under a CC-BY 4.0 International license .

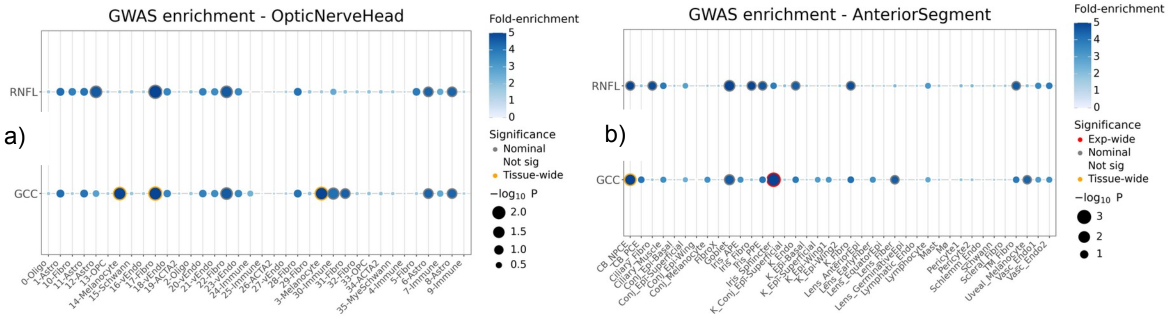

Fig. S2. Cross-ancestry GWAS enrichment in specific cell types in a) optic nerve head, and b) anterior segment using ECLIPSER.

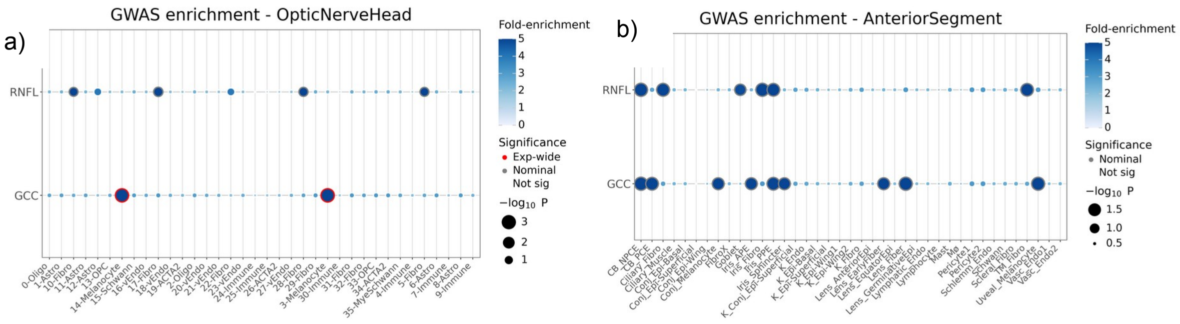

Fig. S3. EUR GWAS enrichment in specific cell types in a) optic nerve head, and b) anterior segment using ECLIPSER.

medRxiv preprint doi: https://doi.org/10.1101/2025.11.04.25339517; this version posted November 6, 2025. The copyright holder for this preprint (which was not certified by peer review) is the author/funder, who has granted medRxiv a license to display the preprint in perpetuity.
It is made available under a CC-BY 4.0 International license .

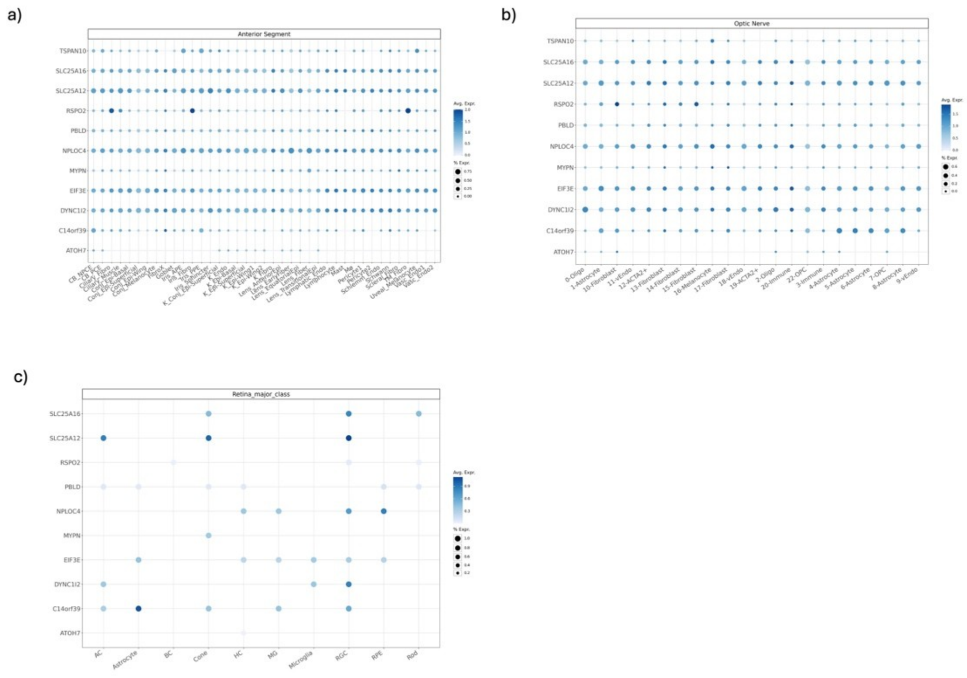

Fig. S4. Single-cell expression of genes in various cell types in a) anterior segment, b) optic nerve head, optic nerve and surrounding posterior tissues, and c) retina. Single nucleus RNA-seq for the anterior segment was taken from van Zyl et al., 2022 *(48)* and for the optic nerve head and surrounding posterior tissues from Monavarfeshani, Yan, *et al.,* 2023 *(49)*.
